# Dietary Intake of Vitamin B12 is Better for Restoring a Low B12 Status Than a Daily High-Dose Vitamin Pill: An Experimental Study in Rats

**DOI:** 10.3390/nu10081096

**Published:** 2018-08-15

**Authors:** Eva Greibe, Ole Nymark, Sergey N. Fedosov, Christian W. Heegaard, Ebba Nexo

**Affiliations:** 1 Department of Clinical Biochemistry, Aarhus University Hospital, Palle Juul-Jensens Boulevard 99, 8200 Aarhus N, Denmark; au335591@post.au.dk (O.N.); e.nexo@dadlnet.dk (E.N.); 2 Department of Molecular Biology and Genetics, Aarhus University, Gustav Wieds Vej 10, 8000 Aarhus C, Denmark; SNFedosov1960@gmail.com (S.N.F.); cwh@mbg.au.dk (C.W.H.)

**Keywords:** dietary vitamin B12, vitamin pills, hydroxocobalamin, cyanocobalamin, tissue distribution, B12-depleted rats, vegan

## Abstract

Vitamin B12 (B12) is present in foods of animal origin, and vegans are encouraged to take supplements with synthetic B12 in order to ensure a sufficient uptake. Recent rat studies suggest that natural (hydroxo-B12, HO-B12) and synthetic (cyano-B12, CN-B12) B12 behave differently in the body. Here, we test if a daily vitamin pill matches dietary B12 in ability to restore a low B12 status in rats. B12-depleted male Wistar rats (*n* = 60) were divided into five groups (*n* = 12 in each) and subjected to two weeks intervention with various schemes of B12 supplementation. Two “dietary” groups received a low-B12 chow that was fortified with either HO-B12 or CN-B12 providing a continuous supply. Two “pill” groups received a single daily dose of CN-B12, where the vitamin content either matched or exceeded by factor four the provisions for the “dietary” groups. A control group received the low-B12 chow without B12 fortification. B12 was measured in plasma and tissues. Dietary B12 provides 35% more B12 to the tissues than an equivalent single daily dose (*p* < 0.0001). Natural B12 delivers 25% more B12 to the liver than synthetic B12 (*p* = 0.0007). A fourfold increase in B12, supplemented as a single daily dose, does not provide any extra B12 to the tissues (*p* = 0.45). We conclude that dietary B12 is better at rescuing a low B12 status than a daily vitamin pill.

## 1. Introduction

Vitamin B12 (B12, cobalamin) is a driving force. It helps to make DNA and blood cells, and it is crucial to sustain a healthy brain and nervous system [1]. The vitamin in its natural form is only present in foods of animal origin, such as milk, meat, and fish, and a well-balanced omnivorous diet is necessary to supply the recommended amount in humans (2.4 µg B12/day) [1]. This poses a challenge for the growing number of vegans worldwide. In most countries, vegans are encouraged to take daily B12 supplements to stay healthy. Traditionally, oral supplements contain 2.4–10 µg of synthetic cyano-B12 (CN-B12), and the manufactures recommends one pill per day. In comparison, food contains natural B12 forms, such as hydroxo-B12 (HO-B12) [2], and it is ordinarily consumed repeatedly during the day. This distinction is important as the intestinal B12 absorptive system is saturated at 1–2 µg B12 per meal [3,4]. Besides being used in oral supplements, CN-B12 is also used for food fortification purposes. The two forms of B12 (in their free form) are equally well absorbed in both man and rat [5,6,7], but recent rat studies suggest that HO-B12 is a superior B12 source for the tissues when compared with CN-B12, as it provides more active B12 coenzymes that are necessary for the B12 metabolism [8].

In the light of these new findings and the growing number of vegans that are relying solely on supplementation with CN-B12, it is important to test whether vitamin pills and dietary B12 supply the body with B12 equally effective for restoring B12 status. Topical problems cover physiological differences in B12 forms, their doses, and the frequency of administration. Here, we investigate the effect of dietary B12 (provided via multiple intakes) and a single daily vitamin pill for two weeks in a B12-depleted rat model. Our aim was to compare vitamin pills (CN-B12), fortified food (CN-B12), and a proxy for natural food (food fortified with HO-B12). We address the following questions: (1) Are dietary HO-B12 (“real food”) and dietary CN-B12 (“fortified food”) equivalent sources of B12? (2) Can a single vitamin pill per day provide the same amount of B12 as multiple dietary intakes? (3) What can be gained by applying a higher B12 content in vitamin pills compared with a lower dose?

## 2. Materials and Methods 

### 2.1. Animals

Male Wistar rats (RjHan:WI) (*n* = 60) from Janvier Labs, Le Genest-Saint-Isle, France, were used for the experiment (seven weeks old; weighing approx. 200 g upon arrival to the animal facility). The study was authorized by the Danish Animal Experimental Inspectorate in agreement with the European Union (EU) directive 2010/63/EU on animal experiments (approval number: 2016-15-0201-00984) and conducted at the animal facility at Faculty of Health, Aarhus University, Denmark. The institutional and national guidelines for care and use of animals were followed, and the rats were checked daily for any health or welfare problems. No signs of pain, suffering, or distress were observed before or during the study.

The rats were housed in pairs in standard cages (Makrolon 1291 H type III H, 800 cm^2^, Techniplast, Buguggiate, Italy) in a controlled environment (20.0 ± 0.5 °C; 60% humidity) with a 12-hour light-12 hour dark cycle. Bedding material (asp chips, Tapvei, Finland) and soft paper wool (LBS biotech, Surrey, UK) were changed daily. The rats were allowed one week of acclimatization in the animal facility before the experiment was initiated. During this time, the rats were kept on a standard stock rat fodder (Altromin 1324, Brogaarden, Lynge, Denmark) containing 24 µg/kg diet CN-B12 (according to the manufacturer) and had free access to tap water. The rats weighed 219 (190–273) g (median and (range)) (*n* = 60) at the beginning of the study.

### 2.2. Study Design

**Figure 1:**
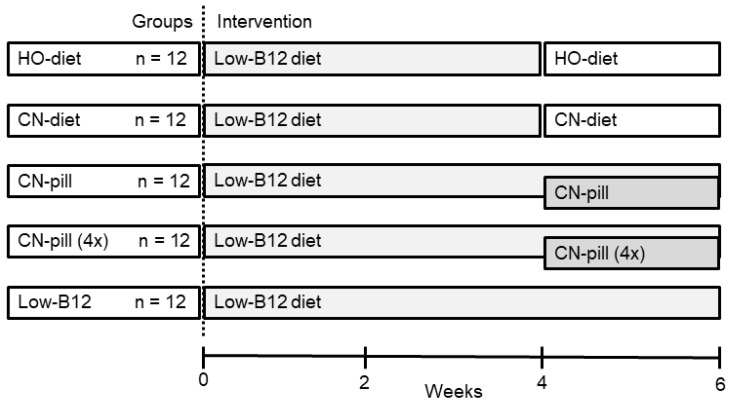
Study design. Male Wistar rats (*n* = 60) received a low-B12 diet for four weeks, followed by two-weeks intervention with B12 in the form of custom-made diets with added B12 (hydroxo-diet (HO-diet), 0.72 µg B12/day; cyano-diet (CN-diet), 0.72 µg B12/day) or a single daily B12 dose embedded in a jelly bean (mimicking a daily vitamin pill) (CN-pill, 0.72 µg B12/day; and, CN-pill (4×), 2.88 µg B12/day). A control group (Low-B12) continued on the low-B12 diet for the six weeks duration of the study.

The experiment was conducted over a period of six weeks (study design is outlined in Figure 1). The 60 rats were fed a low-B12 diet for four weeks in order to obtain a suboptimal B12 status. At this point, the rats were randomly allocated to five groups (“HO-diet”, “CN-diet”, “CN-pill”, “CN-pill (4×)”, “low-B12”, *n* = 12 in each) and were fed a B12-holding diet or a daily vitamin pill with B12 for two additional weeks as outlined in Figure 1. The “low-B12” group continued on the low-B12 diet for the two weeks in question. The dietary groups changed to custom-made diets with added HO-B12 (“HO-diet” group, mimicking natural foods) or CN-B12 (“CN-diet” group, mimicking fortified foods), each of them providing ~0.72 µg HO/CN-B12 per day. The “CN-pill” and “CN-pill (4×)” groups also continued on the low-B12 diet for the two weeks, but received a daily jelly bean (mimicking a vitamin pill) with 0.72 µg CN-B12 or 2.88 µg CN-B12, respectively. For details on rat diets and jellybeans, see Section 2.3 and Section 2.4.

Four times during the experiment (baseline, week 4, week 5, and week 6), the rats were weighed and blood samples were drawn by puncture of the sublingual vein with a 23-gauge needle. After the end of the study (week 6), the rats were anesthetized with isoflurane gas and sacrificed by cervical dislocation. Liver, kidneys, brain (cerebrum), and muscle (thigh) were collected, weighed, and snap-frozen in liquid nitrogen before stored at −80˚C until further processing (see Section 2.6).

### 2.3. B12 Contents of Diets 

All of the rats had free access to food and water throughout the study. The low-B12 diet was purchased commercially (Altromin C1024, Brogaarden, Lynge, Denmark) and indicated to contain <11 µg/kg B12. The diets with HO-B12 and CN-B12 were custom-made by Brogaarden by adding an aqueous solution of crystalline HO-B12 (24 µg/kg diet) or CN-B12 (24 µg/kg diet) to the low-B12 diet. The solutions contained HO-B12 (Vibeden, Sandox, Copenhagen, Denmark) or CN-B12 (Betolvex, Actavis, Gentofte, Denmark) dissolved in demineralized water. Both samples were centrifuged for five minutes at 12,000× *g* at room temperature before retaining the supernatant. Concentrations of B12 were determined by diluting six aliquots 1:10 and converting the B12 form (HO-B12 or CN-B12) to diCN-B12 by incubation with 0.2 M of KCN for one hour in the dark. The concentration of diCN-B12 was determined by measuring the absorbance at 368 nm on the Shimadzu UV-1800 spectrophotometer (Holm & Halby, Broendby, Denmark) and employing the molar absorption coefficient of diCN-B12 (ε_368_ = 30,400 M^−1^ cm^−1^) [9]. The mean of the six stocks was used to calculate the stock volume that is needed to make diets with 24 µg/kg of HO-B12/CN-B12. As the diets with HO-B12 and CN-B12 were made by the addition of free HO-B12 and CN-B12 to the same stock of low-B12 Altromin C-1024 diet, the three diets differed only in the content and the form of the added B12. For quality check, we analysed the forms of B12 in the three diets by in-house HPLC analyses, as previously described [8]. A preliminary estimate indicated that the rats consumed 30 g of food per day, corresponding to an intake of ~0.72 µg HO/CN-B12 per day. This estimate was used for preparation of the B12-containing jelly beans prior to the study (see Section 2.4). The amount (weight) of food consumed during the study was calculated by subtracting the weight of left-overs from the ration provided each day.

### 2.4. Jelly Beans with B12

**Figure 2:**
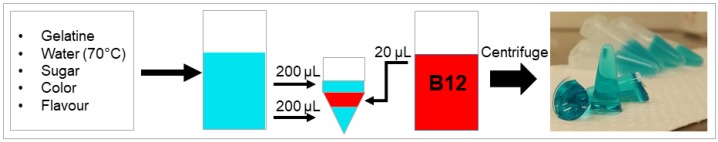
Jelly beans with CN-B12. To mimic a daily vitamin pill, B12 was embedded in a jelly matrix. The rats in the two supplementation groups, “CN-pill” (0.72 µg CN-B12/day) and “CN-pill (4×)” (2.88 µg CN-B12/day), received one B12-containing jelly bean per day for two weeks (from week 4 to week 6). The procedure for preparation of the jelly beans is outlined in details in the method section.

Two batches of 200 jelly beans were produced for the study. One batch of jelly beans contained 0.72 µg CN-B12/jelly bean (“CN-pill” group), corresponding to the approximate daily intake of B12 from the HO-B12 and CN-B12 diets (see Section 2.3). The other batch of jelly beans contained 2.88 µg CN-B12/jelly bean (“CN-pill (4×)” group) corresponding to a fourfold higher B12 dose per day. The recipe for preparing 25 jelly beans is outlined, as follows. Each batch of jelly beans was produced by upscaling this recipe by a factor eight. Gelatine (1.5 g) from porcine skin (Sigma-Aldrich, G2500-500G, gel strength 300) was dissolved in 10 mL demineralized water preheated to 70 °C. To dissolve the gelatin without risking premature solidification of the jelly, the gelatin-water mixture was submerged in a 45 °C water bath and carefully stirred with a spatula for a few minutes until the gelatin was fully dissolved to a homogenous solution. Then, 0.1 g of commercially available sugar (DAN Sukker, Copenhagen, Denmark), 100 µL of raspberry flavor (Tørsleffs, Hvidovre, Denmark), and 150 µL of food color (Tørsleffs, Hvidovre, Denmark) were added and gently mixed into the solution. The flavor was added to give the rats incentive to eat the jelly beans. The color was added to help the animal care takers identify any possible not-eaten jelly beans that were buried in the cage bedding material, and to prevent a mix-up of jelly beans with 0.72 µg CN-B12 (blue) and 2.88 µg CN-B12 (red). Preliminary testing revealed that rats showed a strong partiality for raspberry flavor, but had no color preference (data not shown). Now, 200 µL of the jelly matrix was poured into 1.5 mL Eppendorf tubes, followed by the addition of 20 µL CN-B12 solution (see below), and again followed by addition of another 200 µL of the jelly matrix. The mixture was vortexed for five seconds and centrifuged at 13,000 rpm for two minutes at room temperature to settle the jelly matrix at the bottom on the Eppendorf tube. The tubes were then placed in an up-right position at room temperature for 15 minutes for solidification of the jelly matrix. Finally, the tubes were transferred to the refrigerator and stored at 4 °C for up to two weeks before administration to the rats. At this point, the solidified jelly was removed from the Eppendorf tubes using a spatula giving rise to cone-shaped B12-containing jelly beans (shown in Figure 2). Jelly beans without B12 were prepared in the same manner but omitting B12. They were used for training purposes (see Section 2.5).

The CN-B12 for preparation of the jelly beans originated from the same stock solution as the one added to the CN-diet (see Section 2.3). For the “CN-pill” group (0.72 µg CN-B12/jelly bean), the stock was diluted in sterile water to a concentration of 0.036 µg/µL CN-B12 before addition to the jelly matrix. For the “CN-pill (4×)” group (2.88 µg/jelly bean), the stock was diluted to a concentration of 0.114 µg/µL CN-B12.

### 2.5. Training of Rats to Eat Jelly Beans

Rats are naturally reluctant when it comes to novel foods. Therefore, the rats in the two supplementation groups were trained with placebo jelly beans (without B12) for two weeks (from week 2 to week 4) prior to the two-week intervention period (week 4 to week 6) to overcome this neophobia. During the daily training sessions, the two rats in each cage were separate in two individual cages. On the first day, an empty petri dish was placed in each rat cage. In the following five days, each rat received a petri dish with two jelly beans in the morning and two in the afternoon. For the following eight days, this was reduced to one jelly bean twice a day. In the beginning, the rats exerted great carefulness or even inert avoidance toward the placebo jelly beans, but after the two-week training period, all of the rats were accustomed to the new food and ingested the jelly beans within two minutes after administration. After the 14-days training period (week 2 to week 4), the experiment was conducted in accordance with the study design. Each day the two rats in each cage were separated in individual cages to make sure that each rat consumed one—and only one—jelly bean per day. The rats showed great compliance (willingness to eat the jelly beans) in the study, each ingesting more than 98% of the B12-containing jelly beans administered.

### 2.6. Determination of B12 in Rat Plasma and Tissues

Blood samples were collected into 4 mL lithium heparin tubes (BD Vacutainer), and plasma was removed after centrifugation at room temperature for nine minutes at 1850× *g* and stored at –20 °C until analysis. Plasma was measured for B12 content on the Advia Centaur CP Immunoassay System (Siemens, Erlangen, Germany).

The tissues were thawed on ice and endogenous B12 was extracted from liver, kidneys, brain, and muscle by homogenizing 0.2 g of tissue in 750 mL of Na-acetate buffer (0.4 mol/L, pH 4.4) while using the Precellys 24 (Bertin Technologies, Montigny-le-Bretonneux, France) with three centrifugation cycles of 20 s at 6800 rpm with 30 s pauses between cycles. After homogenization, 20 µL of KCN solution (30 mmol/L) was added to convert all B12 in the samples to CN-B12. Then, the mixtures were boiled for 10 min and centrifuged for 40 min at 20,000× *g* and 4 °C, and the supernatants were collected and stored at –20 °C until analysed. The supernatants were measured for total B12 content on the Advia Centaur CP Immunoassay System (Siemens) after dilution with 0.9% solution of NaCl. The following dilutions were used: liver (1:15, all groups), kidneys (1:100 for the “low-B12” group, 1:300 for the remaining groups), brain (1:5, all groups), and muscle (1:4, all groups). The dilutions were chosen to ensure that the B12 concentrations would be within the range of measurements (100–1476 pmol/L) of the Advia Centaur CP Immunoassay system.

### 2.7. A Pilot Study to Test the Absorption of B12 from Jelly Beans

**Figure A1:**
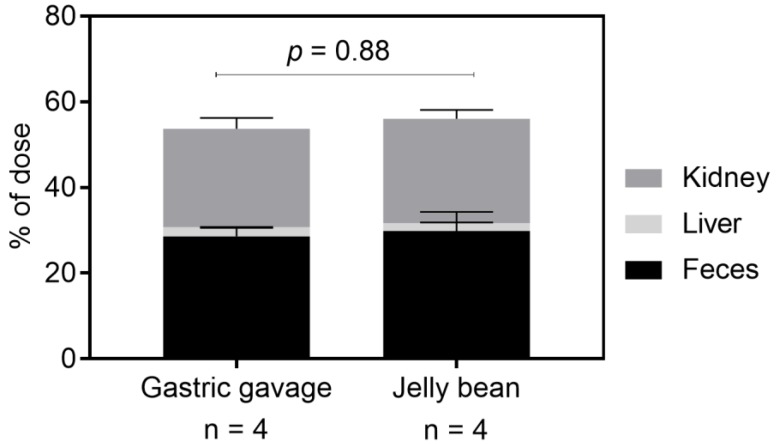
Result of the pilot study. [^57^Co]CN-B12 accumulation 24 hours after oral administration. Kidney, liver, and feces were collected from two groups of rats (*n* = 4 in each group) kept for two weeks on a low-B12 diet before given a single oral dose of radiolabeled [^57^Co]CN-B12 by either gastric gavage or in a jelly bean. Results are given as the percentage of cpm of the given dose of [^57^Co]CN-B12. Stacked mean values with SEM are shown. No differences were observed in kidneys (*p* = 0.69), liver (*p* = 0.34), or feces (*p* = 0.69) between the two groups, or in the sum of cpm measured (*p* = 0.88). The Mann Whitney test was used to compare the two groups, and *p*-values ≤ 0.05 were considered statistical significant.

We tested whether jelly influenced the absorption of B12 by comparing the uptake of labeled B12 being administered either with jelly beans or by gastric gavage, a technique where a tube is used to deliver an oral dose directly into the stomach via the esophagus. Female Wistar rats (*n* = 8) weighing (median (range)) 278 (250–310) g were used in the pilot study. The rats were purchased, housed, and monitored, as explained in Section 2.1, and the study was conducted under the same guidelines and ethical approvals. The rats were kept on the low-B12 diet (see Section 2.3) for two weeks during which time they were simultaneously trained to eat jelly beans (see Section 2.5). On day 15, the rats received a single oral dose of 1 pmol radioactive labeled [^57^Co]CN-B12 (approx. 130,000 cpm with 1.75 µCi/mL CN-B12) (MP Biomedicals, Ohio, USA, catalogue no. 06B-430000) by either gastric gavage (*n* = 4) or in a jelly bean (*n* = 4). For gastric gavage, we used a 20-gauge needle to administer the oral dose of [^57^Co]CN-B12 in 0.5 mL water. The jelly beans were produced, as described in Section 2.4, but with addition of [^57^Co]CN-B12 to the jelly matrix instead of unlabeled B12. Following the oral dose (gastric gavage or jelly bean), the rats were transferred to separate metabolic cages (Scanbur 3700M061, Techniplast, Buguggiate, Italy) for collection of feces. After 24 hours, the rats were sacrificed, and liver and kidneys were harvested and frozen in liquid nitrogen. [^57^Co]CN-B12 was measured by gamma counting using the Wizard Automatic Gamma Counter (Perkin Elmer). In practice, liver and kidneys were thawed on ice, cut into smaller pieces, and transferred to tubes for the gamma counter. All of the tubes were counted to obtain the whole-organ cpm. Results are expressed as the fraction (%) of total administered dose (cpm) of [^57^Co]CN-B12 per organ (Appendix A: Figure A1).

### 2.8. Statistical Analysis

Sample size calculations were used to determine the number of rats in each group. We knew neither the mean nor the standard deviation for changes in plasma B12 of B12 depleted rats in response to intake of B12 vitamin pills for two weeks. We therefore based our sample size calculations on an earlier study, showing a 40% increase in plasma B12 in rats on dietary HO-B12 for two weeks when compared with rats on a low-B12 diet [8]. As we expected, the effect of vitamin pills on plasma B12 to be lower than dietary intake, we chose a minimal relevant difference of 35%. To achieve a statistical power of 90% and a two-sided significance level of 5%, we had to include 10 rats in each group to identify the chosen minimally relevant difference. To account for possible outliers and sick animals, we included 12 rats in each group.

The D’Agostino-Pearson omnibus test was used to determine if the data followed the Gaussian distribution. Some observations were not normally distributed and logarithmic transformation was used to obtain this. Data were analyzed using repeated measurements analysis of variance (ANOVA) (plasma B12 over time) and ordinary one-way ANOVA (group comparisons). For both ANOVA methods, Tukey’s post hoc test was used to correct for multiple comparisons. For some data (dietary intake (g), brain weight (g), kidney B12 (pmol/g)), normality could not be achieved by logarithmic transformation. In these cases, comparisons between groups were made using the Kruskal-Wallis test with Dunn’s corrections. For the pilot study, the two groups (gastric gavage vs. jelly bean) were compared with the Mann Whitney test. As not all of the observations were normally distributed (or could be transformed to a Gaussian shape), the data are described as median with range in the text and tables. Values of *p* ≤ 0.05 were accepted as statistically significant. The data analysis was performed while using the statistical software available in GraphPad Prism version 7.03 (GraphPad, La Jolla, CA, USA).

## 3. Results

We present data on 60 rats kept on a low-B12 diet for four weeks prior to two weeks intervention with either dietary B12 (two groups) or a daily vitamin pill (two groups) or continuing on the low-B12 diet (one group). The dietary groups (“HO-diet” and “CN-diet”) received food with added HO-B12/CN-B12, while the supplementation groups (“CN-pill” and “CN-pill (4×)”) received a single daily dose of CN-B12 that was embedded in jelly. For the “CN-pill” group, the jelly beans contained the same B12 amount as the pre-estimated daily intake of B12 from these diets (0.72 µg/day, see method section). In the pilot study, we showed that B12 was equally well absorbed when being administered in water and in a jelly matrix (Appendix A: Figure A1). 

### 3.1. Consumption of Diets and B12 Intakes

The rat groups had a daily dietary intake of (median (range)) 22.2 (17.4–27.2) g/rat/day (“HO-diet”), 22.0 (18.2–31.7) g/rat/day (“CN-diet”), 21.8 (17.5–27.5) g/rat/day (“CN-pill”), 22.2 (18.8–26.6) g/rat/day (“CN-pill (4×)”), and 22.6 (16.6–29.7) g/rat/day (“low-B12”). There was no difference in food that was consumed per day between the five groups (*p* = 0.43). On average, the 60 rats consumed 22.1 g of the food per day, which was lower than the pre-estimated amount (30 g/day/rat). Consequently, the two dietary groups received ~0.53 µg HO-B12/CN-B12 each day from the food (24 µg B12/kg diet), while the supplementation groups received either 0.72 µg CN-B12 (“CN-pill” group) or 2.88 µg CN-B12 (“CN-pill (4×)” group) from the jelly beans. There was no difference in organ (liver, kidney, brain) weights or total body weights between the rats at the end of the study (Table 1).

### 3.2. Plasma and Tissue B12 after Intervention with Dietary B12 or Vitamin Pills

**Figure 3:**
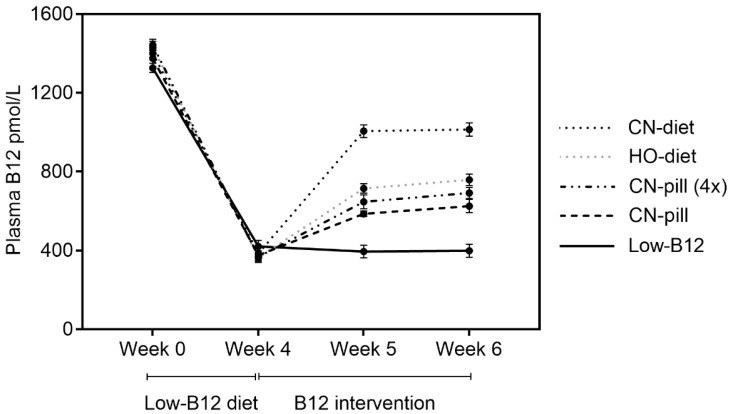
Plasma B12 before and after B12 intervention. Results are shown as means with standard error of mean (SEM).

We measured plasma B12 at baseline (week 0) and after four weeks on a low-B12 diet (week 4), and again after one (week 5) and two (week 6) weeks of B12 intervention. The results are shown in Figure 3. Overall, we found a 73% decline (from 1388 pmol/L to 378 pmol/L (medians)) in plasma B12 in response to the four weeks on low-B12 diet (week 0 vs. week 4, *n* = 60). After this point, the intervention strategies presented different effects on plasma B12. Dietary CN-B12 (“CN-diet”) showed a higher accumulation of B12 in the plasma than both equimolar dietary HO-B12 (“HO-diet”) (week 6, *p* < 0.0001) and CN-B12 in vitamin pills (Figure 3). For the latter, this was the case for both equimolar B12 doses (“CN-pill”) (week 6, *p* < 0.0001) and for vitamin pills with a four times higher B12 concentration (“CN-pill (4×)”) (week 6, *p* < 0.0001). An increase of the CN-B12 in the vitamin pills by a factor of four did not further improve plasma B12 concentrations (“CN-pill” vs. “CN-pill (4×)”, week 6, *p* = 0.56) (Figure 3). Despite the increase in plasma B12 in response to the B12 treatments, none of the groups reached the baseline concentrations within the two week intervention period.

**Figure 4:**
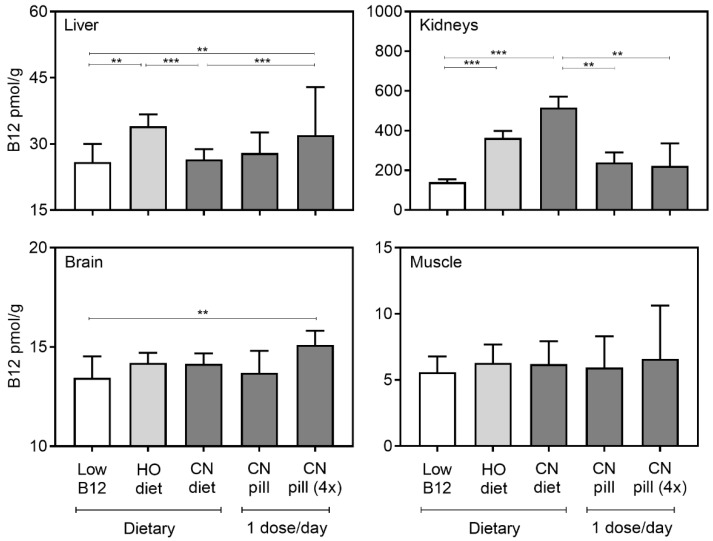
B12 tissue distribution per gram tissues. Liver, kidneys, brain, and muscle tissues were harvested and measured for content of B12. The results are given as pmol/g wet tissue (median with interquartile range). The one-way analysis of variance (ANOVA) with Tukey’s post hoc corrections for multiple comparisons (liver, brain, muscle; normalized data) or the Kruskal-Wallis test with Dunn’s corrections (kidney; not normalized data) was used to compare groups. Asterisks indicate the level of statistical significance (*p* < 0.0005 (***), *p* < 0.005 (**)).

Liver, kidneys, brain, and muscle biopsies were measured for their content of B12. The results are reported as pmol of B12/g of wet tissue in Figure 4. We found that the HO-diet provided more B12 to the liver than the CN-diet, but no differences were observed for the other tissues that were examined. We did see a trend towards a higher kidney B12 for the “CN-diet” group, but this observation was not statistical significant. When compared with vitamin pills, dietary CN-B12 provided more than twice the amount of B12 to the kidneys, even when the pills contained a four times higher daily B12 dose than the diet (Figure 4). In fact, a fourfold increase of the B12 dose in the pills did not provide extra B12 to any tissue. However, we did find that the higher B12 dose in the vitamin pills (“CN-pill (4×)” group) caused a higher accumulation of B12 in the liver when compared with the “CN-diet”. In the brain, a small difference was observed between the “low-B12” group and the high-dose vitamin pill (“CN-pill (4×)”) (*p* = 0.006). No differences in muscle B12 were observed between any groups (Figure 4). We also compared the groups for total content of B12 per organ (pmol) (Table 1), however, the outcome was the same as in Figure 4. The HO-diet provided more B12 to the liver than the CN-diet, and a strong trend to the higher accumulation of CN-B12 in the kidney as compared with HO-B12 was observed. When adjusting for multiple comparison the trend came out insignificant (*p* = 0.469).

**Figure 5:**
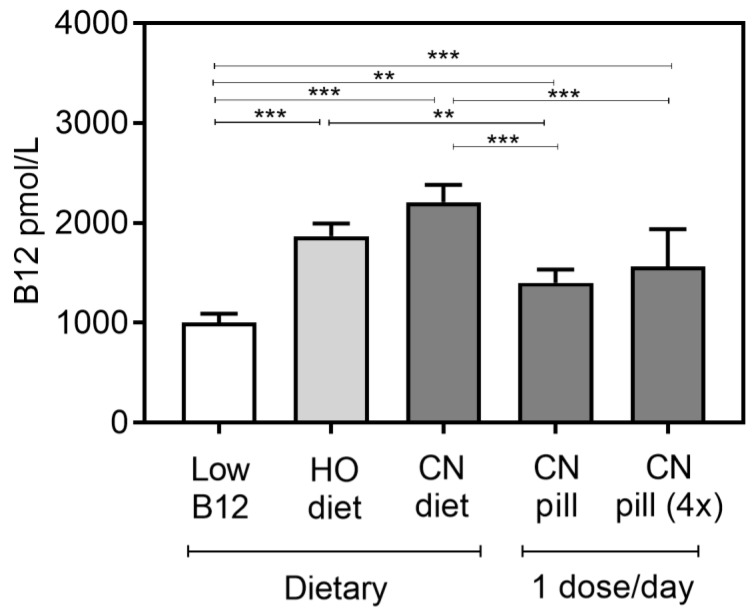
The sum of all B12 recovered in liver, kidney, and brain. Results are shown as median with interquartile range. Tissues were measured for content of B12 and whole-organ B12 was calculated from the B12 content (pmol/g of wet tissues) and the total organ weights. Muscle was not included, as we did not measure the total muscle mass. The one-way ANOVA with Tukey’s post hoc corrections for multiple comparisons was used to compare groups. Asterisks indicate the level of statistical significance (*p* < 0.0005 (***), *p* < 0.005 (**)). The tissue B12 contents between the five groups (B12 per gram tissue) are compared in Figure 4.

The sum of all B12 recovered in liver, kidney, and brain are compared in Figure 5. The total amount of B12 accumulated in the three tissues did not differ significantly between the diet groups, but it was significantly higher than in the pill groups. A single high-dose CN-pill per day did not provide more B12 to the tissues than a daily vitamin pill with a four times lower dose (Figure 5).

## 4. Discussion

Here, we investigate the effect of dietary B12 (continuous intake) and a daily vitamin pill for two weeks in a B12-depleted rat model. We also compare the effect of dietary HO-B12 (a natural form found in food) and CN-B12 (the synthetic form used in vitamin pills and fortified foods), and of normal vs. high-dose vitamin pills. We report three major findings: (1) Dietary B12 is better at restoring B12 status as compared with a daily vitamin pill; (2) Dietary HO-B12 provides more B12 to the liver than dietary CN-B12, in agreement with previous rapports [7,8,10]; and, (3) High-dose vitamin pills do not provide more B12 to the body than normal-dose vitamin pills.

We introduced B12 depletion in the rats by keeping them on a low-B12 diet for four weeks prior to the two weeks intervention with dietary or vitamin pill B12. Endogenous plasma B12 confirmed the B12 depletion at week 4. The control group (“low-B12”), which continued on the low-B12 diet for the six weeks duration of the study, showed a substantial B12 depletion in the kidney (week 6), a known storage organ of B12 in the rat [11,12,13]. However, in liver, brain, and muscle tissues, the depletion was less pronounced. These findings are in agreement with previous rat studies [8,10] and confirm the conjecture that the kidneys store B12 during times of B12 surplus, but recirculate the vitamin for the utilization in the rest of the body during times of B12 deprivation [11,12].

In the study, we used jelly beans for administration of B12 to the “CN-pill” and “CN-pill (4×)” groups. The use of jelly beans for drug delivery in animal studies is becoming increasingly popular in Europe as the “Legislation for the protection of animals used for scientific purposes” (Directive 2010/63/EU) requires the development of new techniques to enhance animal welfare. Whenever possible, voluntary oral administration (adding drugs to drinking water, honey, jelly beans, etc.) should replace forced oral administration (such as gastric gavage, where a metal tube is inserted into the stomach via the esophagus). To the best of our knowledge, we are the first to use jelly beans for oral B12 administration in rats. We therefore conducted a small pilot study to test if B12 absorption is influenced by the jelly matrix. We found no difference in B12 absorption if B12 was administered by gastric gavage or in a single jelly bean. The findings suggest that the jelly matrix does not impair B12 absorption in rats. The results of the pilot study are shown in Appendix A, Figure A1.

We compared the effect of dietary intake of natural HO-B12 (mimicking natural food) and synthetic CN-B12 (mimicking fortified foods) in the rats. We found CN-B12 to provide a much higher B12 accumulation in plasma than dietary HO-B12, whereas the picture was opposite in the liver, which is a key organ for B12-mediated metabolism. This confirms the findings in other rat studies [7,8,10], and suggests that liver cells have a preference for HO-B12 and that CN-B12 has a reduced liver uptake (or an increased excretion). Currently, we do not know if liver cells have a preference for HO-B12 or whether the two forms of B12 are taken up alike, but while HO-B12 is retained in the liver CN-B12 is exported. Further studies are required for clarification of this observation. Besides the liver, no differences in tissue B12 accumulation was observed between the two diets. We have previously shown data suggesting that HO-B12 provides more active B12 coenzymes (5’-deoxyadenosyl-B12) to the tissues [8]. This observation, combined with a higher liver HO-B12 accumulation, indicates that natural foods may be a better source of B12 for the tissues than fortified foods with synthetic B12.

When compared to dietary B12, a daily vitamin pill provided much less B12 to the plasma and tissues. We believe that this difference is caused by a limited absorption capacity for B12 [3,4]. Dietary B12 is supplied frequently during the day and the B12 absorption system is, therefore, constantly supplied with small doses of B12 that are easily taken up by the enterocytes. However, when B12 is administered in a single large dose, as in vitamin pills, the absorption system becomes saturated and only a small additional fraction is taken up by passive diffusion. In humans, around 1% of a given dose is absorbed by passive absorption [14]. In this rat study, we do not see any additional intake from increasing the dose in the vitamin pills by a factor of four.

Our work has some limitations. Prior to the study, we pre-estimated that the rats would consume 30 g of diet per day corresponding to an intake of 0.72 µg B12 per day. We therefore prepared the jelly beans with 0.72 µg CN-B12 (“CN-pill group”) and 2.88 µg CN-B12 (“CN-pill (4×)” group), respectively. However, the rats only consumed 22.1 g of diet per day, which corresponds to a daily intake of ~0.53 µg B12 from the HO/CN-diets (24 µg B12/kg diet). This means that the “CN-pill” group, designed to match the dietary B12 intake, received ~25% more B12 per day than the dietary groups (“HO-diet” and “CN-diet”). Even at the unfavorable odds, we find a better B12 status in the dietary groups when compared with the pill groups. In fact, we may have seen an even greater effect if the dietary groups had received the same daily B12 dose as the pill groups. Interestingly, none of the rats reached baseline plasma B12 concentrations within the two-week intervention period, as expected from a previous rat study with a similar design [8]. This may be explained by the fact that the rats in the present study had a lower plasma B12 after four weeks on a low-B12 diet (median: 378 pmol/L) as compared with the rats in the previous study (median: 558 pmol/L).

Our findings that dietary B12 provides an advantage over vitamin pills may have implications. Especially for vegans, who usually rely on a single daily vitamin pill for B12 supply. Our study suggests the need for human studies to clarify whether food fortification is superior to vitamin pills when it comes to ensuring a sufficient B12 uptake in the vegan population.

## 5. Conclusions

Our studies in rats show that dietary B12 is better at rescuing a low B12 status when compared with a daily vitamin pill, which is possibly due to a limited capacity of the B12 absorption system. This also explains why an increase in the vitamin pill dose did not provide more B12 to the body than vitamin pills with a lower dose. The results highlight the need for human studies to clarify whether food fortification is a better strategy than a daily vitamin pill, when it comes to an improvement of the B12 uptake in the vegan population.

## Figures and Tables

**Table 1:** Total organ B12 contents ^1^.

	Mean Organ Weight (g)	Whole-Organ B12 (pmol)				
	All rats *n* = 60	HO-diet *n* = 12	CN-diet *n* = 12	CN-pill *n* = 12	CN-pill (4×) *n* = 12	Low-B12 *n* = 12
Liver	18.1 (12.5–23.2)	617 (455–813)	478 (350–636)	546 (363–689)	613 (456–844)	489 (429–630)
Kidneys	3.4 (2.7–4)	1229 (701–1588)	1680 (1161–1977)	878 (536–1130)	800 (452–1956)	463 (254–889)
Brain	2.4 (2.1–2.9)	34.8 (30.5–41.3)	34.0 (29.1–38.2)	34.7 (27.7–45.5)	37.7 (31.9–43.2)	32.9 (28.1–40.2)
Body weight (g)	444 (369–540)	444 (369–503)	450 (385–540)	439 (379–531)	438 (411–502)	452 (390–505)

^1^ Results are given as median (range). Whole-organ B12 is calculated from the tissue B12 contents per gram and the total organ weight (g). Whole-muscle B12 is not included in the table, as we did not collect all muscle tissue. The five groups of rats consumed the same amount of rat diet during the study (22.1 g/day).
